# Opioids and Dementia in the Danish Population

**DOI:** 10.1001/jamanetworkopen.2024.45904

**Published:** 2024-11-19

**Authors:** Nelsan Pourhadi, Janet Janbek, Christiane Gasse, Thomas Munk Laursen, Gunhild Waldemar, Christina Jensen-Dahm

**Affiliations:** 1 Danish Dementia Research Centre, Department of Neurology, Copenhagen University Hospital - Rigshospitalet, Copenhagen, Denmark; 2 Department of Affective Disorders, Aarhus University Hospital Psychiatry, Aarhus, Denmark; 3 Department of Clinical Medicine, Aarhus University, Aarhus, Denmark; 4 National Centre for Register-Based Research, Aarhus University, Aarhus, Denmark; 5 Centre for Integrated Register-based Research, Aarhus University, Aarhus, Denmark; 6 Department of Clinical Medicine, University of Copenhagen, Copenhagen, Denmark

## Abstract

**Importance:**

Opioids have been studied as a potential risk factor for dementia, but evidence concerning long-term noncancer opioid use and exclusive use of weak opioids and associated dementia risk is sparse.

**Objective:**

To assess the association between cumulative noncancer use of opioids and risk of age-related all-cause dementia.

**Design, Setting, and Participants:**

This nested case-control study within a population-based cohort included 1 872 854 individuals without previous dementia, cancer, opioid addiction, or opioid use in terminal illness. Data were obtained from national Danish registers. Each individual who developed dementia during follow-up was incidence-density matched to 5 dementia-free controls. Statistical analysis was performed from August 2023 to March 2024.

**Exposure:**

Cumulative opioid exposure was based on filled prescriptions available from 1995 through 2020.

**Main Outcomes and Measures:**

Conditional logistic regression provided adjusted incidence rate ratios (IRRs) for associations between opioids and dementia.

**Results:**

Among 1 872 854 individuals without previous dementia, cancer, opioid addiction, or opioid use in terminal illness included in the study, 93 638 (5.0%) developed all-cause dementia during follow-up (51 469 [55.0%] female; median [IQR] age, 78.1 [73.0-82.8] years) and were matched to 468 190 control individuals (257 345 [55.0%] female; median [IQR] age, 78.0 [73.0-82.8] years). Opioid use up to 90 total standardized doses (TSDs) was not consistently associated with dementia risk. Opioid exposure above 90 TSDs yielded increased IRRs of dementia occurring before age 90 years ranging from 1.29 (95% CI, 1.17-1.42) for 91 to 200 TSDs to 1.59 (95% CI, 1.44-1.76) for greater than 500 TSDs for age-band 60 to 69 years at dementia diagnosis. Corresponding IRRs were 1.16 (95% CI, 1.11-1.22) to 1.49 (95% CI, 1.42-1.57) for age-band 70 to 79 years and 1.08 (95% CI, 1.03-1.14) to 1.21 (95% CI, 1.16-1.27) for 80 to 89 years. Sensitivity analyses corroborated associations in individuals with chronic noncancer pain and with use of weak opioids.

**Conclusions and Relevance:**

This study found that opioid use of less than 90 TSDs was not significantly associated with increased dementia risk. Above 90 TSDs of opioid use was associated with an elevated dementia risk before age 90 years, which persisted in individuals with chronic noncancer pain and in individuals solely exposed to weak opioids. Further research should ascertain whether the findings denote causality between opioids and dementia risk.

## Key Points

**Question:**

Is cumulative use of opioids for noncancer pain associated with increased risk of dementia?

**Findings:**

In this nested case-control study within a population-based cohort including 93 638 individuals with dementia in Denmark, there was no significant association between noncancer opioid use below 90 total standardized doses (TSDs) and dementia risk, whereas opioid use above 90 TSDs was associated with increased dementia risk. This association persisted among individuals with chronic pain diagnoses and with use of weak opioids only.

**Meaning:**

These results suggest that further research is warranted to ascertain the potential link between opioids and dementia.

## Teaser

This nested case-control study within a population-based cohort investigates the association between cumulative noncancer use of opioids and risk of age-related all-cause dementia among Danish adults aged 60 years and older.

## Introduction

Worldwide, dementia is a growing public health challenge with over 10 million new cases annually and a rapidly increasing global prevalence.^1^ Identification of modifiable risk factors is crucial, as the disease remains incurable.^1^ Opioids have been studied as a potential risk factor, but it is currently unknown whether its use increases dementia risk.

Global opioid use has more than doubled from 2000 to 2019.^2^ Studies report of increasing use with advancing age in Europe and the US.^3,4^ Chronic noncancer pain is a highly prevalent condition affecting around 20% of adults.^5^ In Denmark, the prevalence increased from 20% to 28% between 2000 and 2017, with an annual incidence of 2.7%.^6^ Opioids are frequently used to treat chronic noncancer pain, but evidence of the effect on pain management and quality of life is lacking.^7^

Serious adverse effects of opioids include respiratory depression, addiction, and increased mortality.^8^ Although opioid-related neurological adverse effects (eg, altered cognition, sedation, and delirium) are thought to be reversible,^9^ less is known about the long-term safety.^10^ Opioids can affect the brain through neuroinflammation,^11^ and Alzheimer-related brain changes of β-amyloid and tau deposition have been demonstrated postmortem in individuals with opiate use disorder.^12^

Evidence concerning effects of long-term opioid use on cognition and dementia is limited. Only a few previous studies have investigated this research question with inconsistent findings.^13,14,15,16^

A community-based cohort study reported a positive association between the highest cumulative opioid doses and dementia.^13^ A Finnish register-based study found opioid use not to be associated with Alzheimer disease but called for further studies in populations with higher opioid exposure levels.^14^ An Israeli cohort study reported increased dementia risk for opioid use between ages 75 and 80 years,^15^ suggesting a potential critical window of exposure. Additionally, a study reported increased dementia risk in an Asian population with chronic pain.^16^ However, in the latter 2 studies, biases including protopathic bias may have influenced the findings because no lag-time windows were applied between the exposure and outcome. Furthermore, most previous studies were limited by not considering chronic pain related to opioid use, thus they were susceptible to confounding by indication.

Focusing on addressing previous limitations and understudied aspects of the research question, this nationwide population-based study investigated the association between cumulative noncancer opioid use and age-related risk of all-cause dementia. Associations were further assessed in subpopulations with chronic noncancer pain and with use of weak opioids only.

## Methods

### Ethics Approval

According to Danish law, register-based studies are not required to obtain ethical approval or patient consent. This study was approved by the Danish Health Data Authority and the Danish Data Protection Agency. We followed the Strengthening the Reporting of Observational Studies in Epidemiology (STROBE) reporting guideline for cohort studies.^17^

### Study Population and Design

This population-based study used a nested case-control design within a nationwide cohort to assess the associated risk of late-onset all-cause dementia with cumulative noncancer opioid use. We identified an open cohort of individuals aged 60 to 75 years between 2000 and 2020 by linking national Danish registers^18,19,20,21,22^ through an encrypted unique personal identification number assigned to all Danish residents (eTable 1 in Supplement 1). Exclusion criteria were previous history of dementia, opioid addiction, opioid use in terminal illness, or cancer except nonmelanoma skin cancer, the latter due to focusing on noncancer opioid use (eTable 1 in Supplement 1). Included individuals were followed for up to 21 years until dementia, death, emigration, an exclusion criterion, or end of study (December 31, 2020).

Within the cohort, we applied a nested case-control design yielding a matched population of all incident dementia cases during follow-up (2000-2020) and a representative group of noncases/controls sampled from the source cohort. By incidence-density matching (risk set sampling)^23^ on the date of dementia incidence (index date), we matched each case by birth year and sex to 5 eligible, uncensored dementia-free controls. The matched population included unique risk-sets each including 1 dementia case and 5 noncases who were still at risk at index date.^24^

### Dementia

All-cause dementia was defined as dementia of any etiology from age 60 years identified from the first date of dementia diagnosis or filled prescription with antidementia medication (eTable 1 in Supplement 1). In Denmark, dementia is typically diagnosed in hospital memory clinics. Dementia syndrome hospital diagnoses have high validity,^25^ and were provided by The National Patient Register holding data on Danish hospital diagnoses since 1977.^19^ Filled prescriptions with antidementia medication (used for Alzheimer disease, Lewy body dementia, and Parkinson disease dementia) permitted identification of dementia cases diagnosed and treated in primary care.^22^ Age at index date was grouped in 10-year age groups (60-69; 70-79; 80-89; ≥90), as previous studies have found interaction between age at diagnosis and exposure to other pharmacological products.^26^

### Opioid Use

Information on opioid use was drawn from The National Prescription Register providing complete data on all prescriptions redeemed from Danish pharmacies since 1995 (eTable 2 in Supplement 1). Besides the Anatomical Therapeutic Chemical code, prescription data included the date of purchase, number of purchased packages, package size, strength, dosage, and form or mode of administration.

To accommodate differences in potencies and dosages across various opioid formulations and modes of administration, the specific equianalgesic ratio of each opioid subtype was used to calculate the oral morphine equivalent dose (OMEQ) consistent with previous studies (eTable 2 in Supplement 1).^27^ The amount of OMEQ was converted into total standardized doses (TSDs) where 1 TSD corresponds to 30 mg oral morphine equivalents^13,14^ (eTable 2 in Supplement 1).

Dementia neuropathology typically develop gradually over decades, thus, a 5-year lag-time window was consistently applied to address reverse causation/protopathic bias (ie, when early symptoms of dementia lead to opioid use). Consequently, we omitted opioid prescriptions redeemed within 5 years of index.

An individual was considered exposed from first prescription redemption of any opioid between 1995 and the beginning of the lag-time window. The cumulative number of TSDs was categorized similarly as in previous studies: 0 to 30; 31 to 90; 91 to 200; 201 to 500; and more than 500.^28,29^

### Confounders

As potential confounders, we included all available variables known or suspected to be associated with dementia risk and opioid use.^28^ These included sociodemographic confounders; age, sex, and educational level (elementary/secondary school; vocational education; university education). Health-related confounders defined from diagnoses and prescriptions with disease-specific medication included diabetes, hypertension, dyslipidemia, and cardiovascular disease defined by diagnosis of ischemic heart disease or stroke, or prescription with oral antithrombotic medication. The Charlson Comorbidity Index (CCI) was included as a comorbidity burden measure.^29^ Covariates were measured at the beginning of the lag-time window. Exact definitions of variables are displayed in eTable 1 in Supplement 1.

### Statistical Analysis

Conditional logistic regression yielded adjusted incidence rate ratios (IRRs) and 95% CIs for associations between cumulative opioid use and dementia according to age at index.^23^ The reference group was individuals who had not received opioids, and analyses included 5-year lag-time windows.

All models included the potential confounders as covariates; 1.8% had missing information on educational level, hence, subcategorized separately as missing in analyses. Information on the remaining covariates was accessible for all individuals. χ^2^ test provided *P* values for descriptive variables. Two-sided *P* < .05 was considered statistically significant. Data management and analysis was performed from August 2023 to March 2024 using R statistical software version 4.3.2 (R Project for Statistical Computing) and SAS software version 9.4 (SAS Institute).^30^ Incidence-density matching was performed using the R program incidenceMatch from the package tagteam/heaven accessible on Github.

Sensitivity analyses in separate subpopulations addressed potential confounding by indication. First, subpopulation restricted to individuals with chronic noncancer pain^28^ diagnosed within 10 years of study entry to increase the probability of pain-related symptoms and related subsequent opioid use. All individuals had pain-intensive diagnoses, thus aiming to reduce confounding by chronic pain and related conditions. Diagnoses constituted specific pain-intensive back diagnoses, intervertebral disc pain, arthritic pain, posttraumatic fracture pain, and neuropathic pain as seen in previous research (eTable 1 in Supplement 1).^28^ Opioid prescription more than 1 year before the pain diagnosis led to exclusion.

Second, subpopulation restricted to weak opioid use (eTable 2 in Supplement 1) as the exposure of interest. Prescription with strong opioids led to exclusion, thus eliminating potential confounding attributed to strong opioid use. Tramadol is the most frequently used opioid in Denmark and was prescribed at increasing doses over the study period, particularly among age groups younger than 80 years.^31^

Third, subpopulation with indication for opioid use restricted to cough suppressants (ie, opioid antitussives) (eTable 2 in Supplement 1). Prescription with any analgesic opioid led to exclusion, thereby addressing potential confounding by indication from opioids with analgesic indication.

A post hoc sensitivity analysis addressed potential false-positive dementia diagnoses due to reversible opioid-related cognitive impairment by analyzing opioid user status defined according to the date of last filled prescription plus 30 additional days.

To analyze the potential of reverse causation, main analyses were additionally conducted with a shorter 1-year lag-time window for comparison. To analyze indication of potential time-varying effects of confounders, main analyses were additionally conducted with confounders defined at baseline. Finally, we estimated mortality rates among individuals receiving opioids vs those not receiving opioids for indication of potential competing risk of death.

## Results

**Figure 1.**
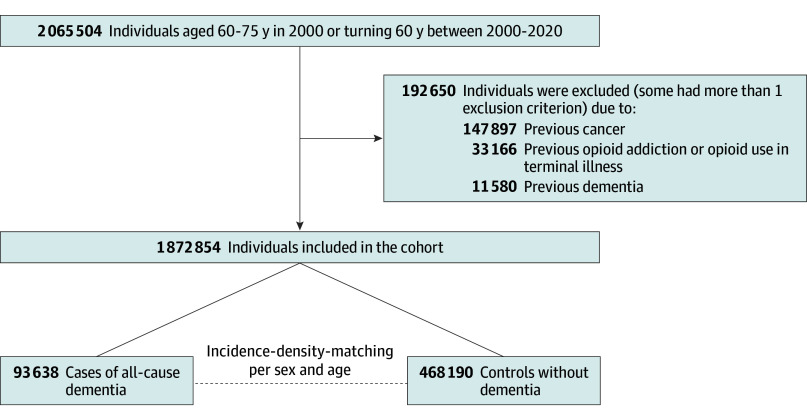
Cohort and Nested Case-Control Population Flowchart During follow-up, 670 200 individuals were censored due to incident cancer (n = 370 407), opioid addiction or opioid use in terminal illness (n = 37 882), emigration (n = 12 011), or death (n = 249 900).

In the nationwide cohort of 1 872 854 individuals, 93 638 (5.0%) developed all-cause dementia during follow-up (51 469 [55.0%] female; median [IQR] age, 78.1 [73.0-82.8] years) and were matched to 468 190 control individuals (257 345 [55.0%] female; median [IQR] age, 78.0 [73.0-82.8] years). Median (IQR) follow-up time was 11.8 (7.3-15.8) years. Figure 1 shows the cohort and nested case-control formation. The median (IQR) age at diagnosis was 78 (73-83) years and 51 469 (55.0%) were female. Overall, comorbidities were more prevalent in dementia cases compared with controls (Table).

**Table.** Characteristics of Nested Case-Control Population

Variable	All-cause dementia (n = 93 638)	Controls (n = 468 190)	*P* value
Sex, No. (%)			
Female	51 469 (55.0)	257 345 (55.0)	>.99
Male	42 169 (45.0)	210 845 (45.0)	
Age at index, median (IQR), y	78.1 (73.0-82.8)	78.0 (73.0-82.8)	.70
Age at index, No. (%), y			
60-69	13 738 (14.7)	68 690 (14.7)	>.99
70-79	42 757 (45.7)	213 785 (45.7)	
80-89	34 929 (37.3)	174 645 (37.3)	
≥90	2214 (2.4)	11 070 (2.4)	
Follow-up time, median (IQR), y	11.8 (7.3-15.8)	11.8 (7.3-15.8)	>.99
Year of index, median (IQR)	2013 (2008-2017)	2013 (2008-2017)	>.99
Opioid ever use, No. (%)	52 232 (55.8)	247 892 (52.9)	<.001
Cumulative opioid use, median (IQR), TSDs^a^	34.8 (13.3-121.8)	32.5 (11.7-94.0)	<.001
Cumulative opioid use, No. (%), TSDs^a^			
0-30	23 818 (25.4)	122 399 (26.1)	<.001
31-90	12 829 (13.7)	62 221 (13.3)	
91-200	5979 (6.4)	26 539 (5.7)	
201-500	4091 (4.4)	16 821 (3.6)	
>500	5515 (5.9)	19 912 (4.3)	
Age at opioid initiation, median (IQR), y^a^	65.5 (59.6-70.2)	65.8 (59.8-70.4)	<.001
Education, No. (%)			
Elementary/secondary school	47 198 (50.4)	228 256 (48.8)	<.001
Vocational education	32 693 (34.9)	164 333 (35.1)	
University education	12 098 (12.9)	67 059 (14.3)	
Missing	1649 (1.8)	8542 (1.8)	
Cardiovascular disease, No. (%)	41 868 (44.7)	182 032 (38.9)	<.001
Stroke, No. (%)	8852 (9.5)	27 384 (5.8)	<.001
Ischemic heart disease, No. (%)	19 063 (20.4)	85 015 (18.2)	<.001
Antithrombotic medication, No. (%)	36 952 (39.5)	161 266 (34.4)	<.001
Diabetes, No. (%)	10 830 (11.6)	40 020 (8.5)	<.001
Hypertension, No. (%)	58 479 (62.5)	274 758 (58.7)	<.001
Dyslipidemia, No. (%)	28 803 (30.8)	130 250 (27.8)	<.001
Charlson Comorbidity Index, No. (%)			
0	81 716 (87.3)	418 681 (89.4)	<.001
1	9696 (10.4)	40 996 (8.8)	
2	1701 (1.8)	6672 (1.4)	
≥3	525 (0.6)	1841 (0.4)	
AIDS/HIV	18 (0.0)	70 (0.0)	.42
Chronic pulmonary disease	4147 (4.4)	17 658 (3.8)	<.001
Heart failure	2289 (2.4)	9456 (2.0)	<.001
Hemiplegia/paraplegia	83 (0.1)	305 (0.1)	.02
Mild liver disease	323 (0.3)	866 (0.2)	<.001
Peptic ulcer disease	1644 (1.8)	5609 (1.2)	<.001
Peripheral vascular disease	2727 (2.9)	11 029 (2.4)	<.001
Kidney disease	638 (0.7)	2617 (0.6)	<.001
Rheumatic disease	1873 (2.0)	8935 (1.9)	.06
Severe liver disease	111 (0.1)	240 (0.1)	<.001

^a^ Numbers relating to opioid use include 5-year lag-time window.

Individuals who had ever received opioids constituted 52 232 dementia cases (55.8%) and 247 892 controls (52.9%). The median (IQR) cumulative opioid use was 35 (13-122) TSDs for cases and 33 (12-94) TSDs for controls. Median (IQR) age at first opioid prescription was 66 (60-70) years. The most-used strong opioids were oral morphine and oxycodone, whereas the most-used weak opioid was oral tramadol. eTable 3 in Supplement 1 displays comprehensive details on opioid formulations and usage.

**Figure 2.**
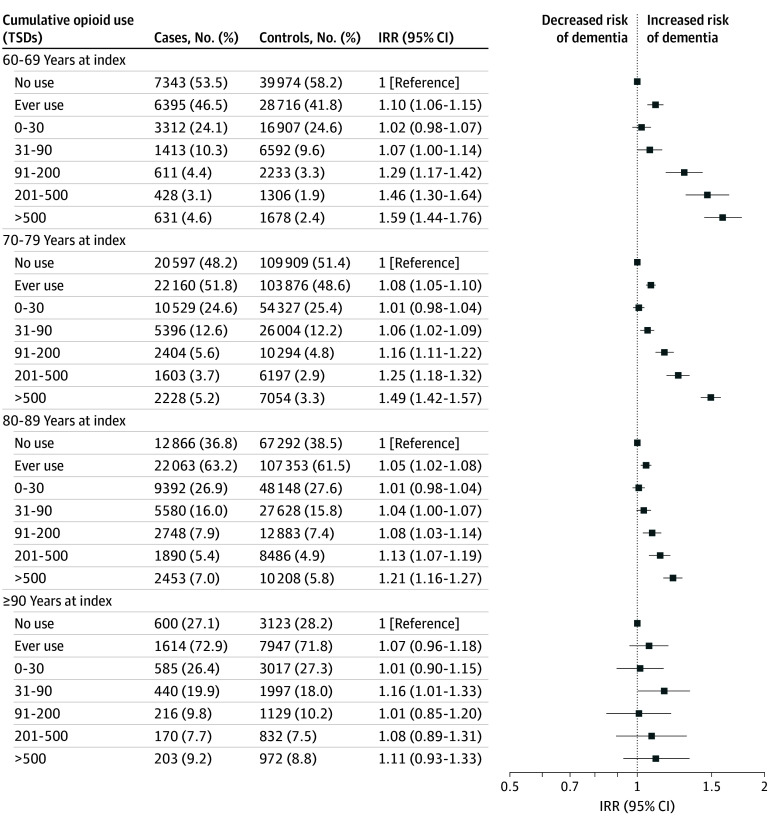
Adjusted Incidence Rate Ratios (IRRs) of Cumulative Opioid Use and All-Cause Dementia According to Age at Index Adjusted for educational level, cardiovascular disease, diabetes, hypertension, dyslipidemia, and Charlson Comorbidity Index score. Five-year lag-time window applied. TSD indicates total standardized daily dose.

Opioid ever use compared with nonuse was associated with a slightly elevated IRR of dementia for 10-year age bands occurring before age 90 (Figure 2). Overall, cumulative opioid exposure of less than or equal to 90 TSDs was not consistently associated with dementia, although, IRRs for 31 to 90 TSDs reached statistical significance for ages 70 to 79 years and at least 90 years at index. Increasing exposure to more than 90 TSDs exhibited increasing IRRs of dementia ranging from 1.29 (95% CI, 1.17-1.42) for 91 to 200 TSDs to 1.59 (95% CI, 1.44-1.76) for greater than 500 TSDs in individuals aged 60 to 69 years at index. Corresponding IRRs for ages 70 to 79 years and 80 to 89 years at index were 1.16 (95% CI, 1.11-1.22) to 1.49 (95% CI, 1.42-1.57) and 1.08 (95% CI, 1.03-1.14) to 1.21 (1.16-1.27), respectively. Opioid use did not show a statistically significant association with dementia occurring at age 90 years or older.

### Sensitivity Analyses

**Figure 3.**
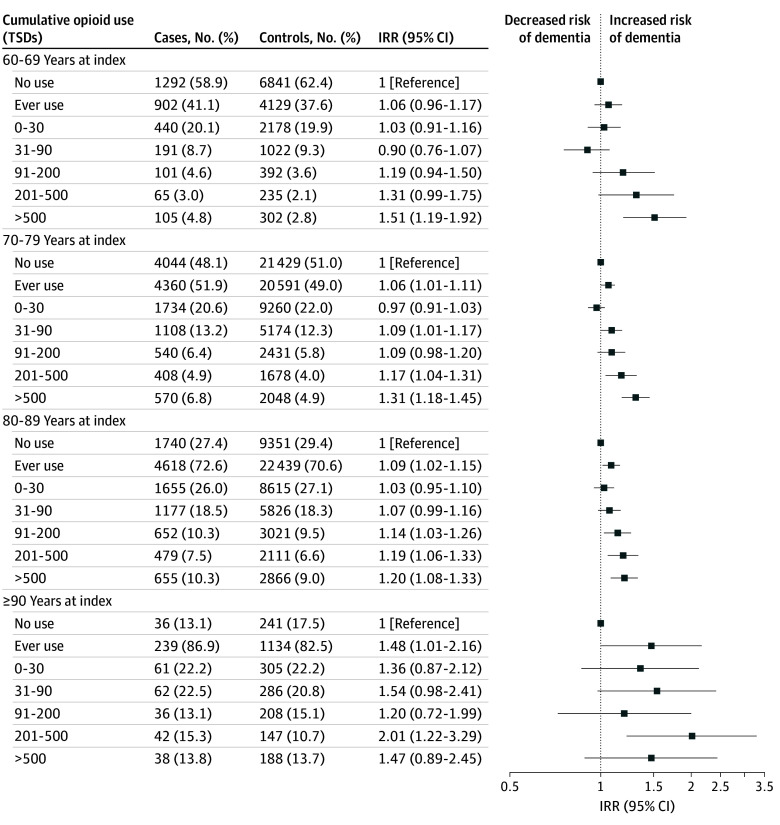
Adjusted Incidence Rate Ratios (IRRs) of Cumulative Opioid Use and All-Cause Dementia According to Age at Index in a Population of Individuals With Chronic Noncancer Pain Diagnosis Adjusted for educational level, cardiovascular disease, diabetes, hypertension, dyslipidemia, and Charlson Comorbidity Index score. Five-year lag-time window applied. TSD indicates total standardized daily dose.

Among individuals with specified chronic noncancer pain, dementia rates decreased compared with the entire study population. Overall, associations persisted for exposure greater than 90 TSDs, although based on fewer cases and with limited statistical precision (Figure 3). Notably, for ages 60 to 69 years at index, opioid ever use did not exhibit a statistically significant association with dementia. This was similarly the case for 91 to 200 TSDs for age bands of 60 to 79 years.

**Figure 4.**
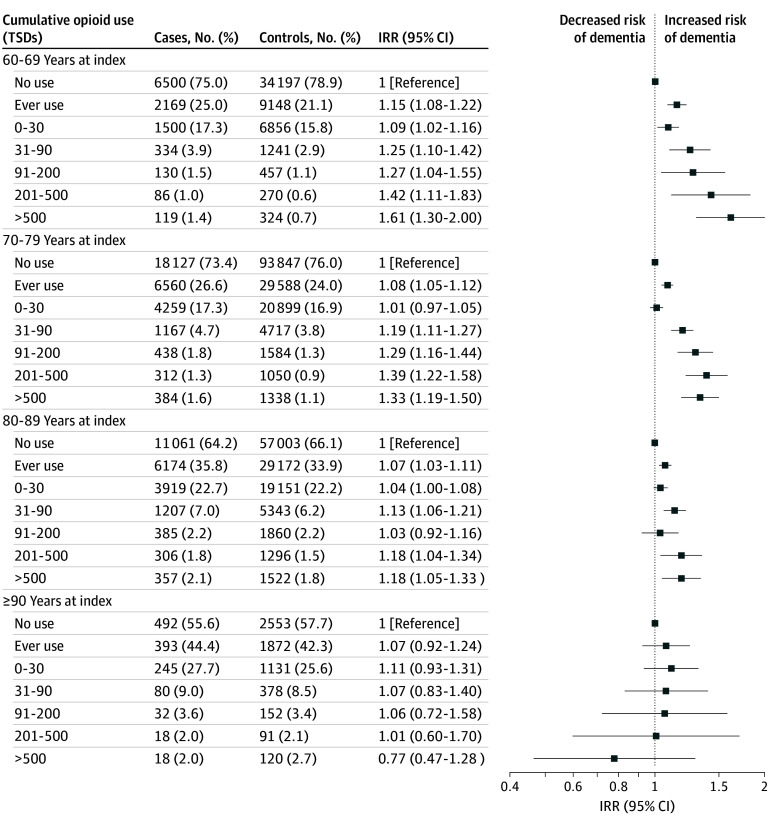
Adjusted Incidence Rate Ratios (IRRs) of Cumulative Use of Weak Opioids and All-Cause Dementia According to Age at Index Adjusted for educational level, cardiovascular disease, diabetes, hypertension, dyslipidemia, and Charlson Comorbidity Index score. Five-year lag-time window applied. TSD indicates total standardized daily dose.

Exclusive use of weak opioids was associated with slightly increased IRRs of dementia before 90 years of age ranging from 1.15 (95% CI, 1.08-1.22) for 60 to 69 years to 1.07 (95% CI, 1.03-1.11) for 80 to 89 years age band (Figure 4). Use of greater than 30 TSDs exhibited elevated dementia rates, except for the category 91 to 200 TSDs for cases aged 80 to 89 years.

Regardless of user status (timing of last treatment day according to index), at least 90 TSDs of opioids was positively associated with dementia before age 90 years (eFigure 1 in Supplement 1). Exclusive use of opioid antitussives was not associated with dementia apart from greater than 200 TSDs for dementia up to age 80 years (eFigure 2 in Supplement 1). Main findings remained largely unchanged in sensitivity analyses with 1-year lag-time and with covariates assessed at baseline (eFigure 3 and eFigure 4 in Supplement 1). Compared with nonuse, opioid use greater than 90 TSDs was associated with increased mortality rate (eFigure 5 in Supplement 1).

## Discussion

In this nationwide study, cumulative opioid use up to 90 TSDs was found not to be uniformly associated with dementia risk. Exposure to more than 90 TSDs of opioids was associated with slightly increased dementia rates primarily before age 90 years at diagnosis. For exposure above 90 TSDs, the overall associations persisted among individuals receiving weak opioids only and among individuals with chronic noncancer pain, although with limited statistical precision.

Our findings align with a recent study reporting elevated dementia risk among individuals receiving opioids in an Asian population with chronic pain.^16^ Additionally, we observed that the increased risk was consistent with opioid use above 90 TSDs and dementia occurring before age 90 years, both in the general cancer-free population and in individuals with pain-intensive diagnoses. Furthermore, we found that associations persisted among individuals solely exposed to weak opioids.

A Finnish registry-based study reported no association between opioid use and Alzheimer disease.^14^ Differences in our findings may be explained by the relatively lower opioid consumption in Finland compared with other Scandinavian countries during the study period.^32,33^ In 2006, the annual average opioid use was 1979 mg OMEQ per Norwegian receiving opioids vs 6025 mg OMEQ per Dane receiving opioids,^32^ making Denmark a relevant setting to investigate high opioid exposure and dementia risk.^32,33^

Furthermore, in our study, exposure history was available for a longer period (maximum 26 years vs maximum 16 years in the Finnish study), and we additionally stratified by age at dementia diagnosis, which interacted with the association exhibiting decreasing rates with advancing age at diagnosis. Dementia in different ages potentially vary in underlying pathology and risk factors. Thus, this observation could represent a critical window of exposure or the diminishing influence of specific risk factors on neuropathology advancing over decades. Finally, low number of cases and unexposed aged greater than 90 years may also have influenced the findings for this age group.

Unexpectedly, among those receiving weak opioids, 31 to 90 TSDs showed positive associations with dementia. Potentially, the exposure intervals of weak opioid use may correspond to longer treatment duration with lower potent doses compared with strong opioids. Whether longer duration of opioid exposure with lower analgesic potency may influence cognition to a larger extent than shorter-term exposure with higher analgesic potency should be investigated in future research.

Previous studies have reported associations between opioid use and cognitive impairment in neuropsychological tests.^34^ Thus, its usage could increase the probability of getting diagnosed with dementia by either demasking clinical dementia symptoms related to underlying neuropathology or by mimicking potentially reversible dementia symptoms. The latter possibility was tackled by applying a 5-year lag-time window and by the sensitivity analysis assessing user status demonstrating increased dementia rates even among individuals finalizing treatment more than 5 years before index.

Opioids cross the blood-brain barrier and target receptors in the central nervous system relating to pain transmission pathways.^35^ Autopsy studies have reported increased deposition of Alzheimer disease related proteins β-amyloid and tau in individuals with high opioid usage and opioid use disorder.^12,36^ Thus, high opioid exposure may influence neurodegeneration and subsequent cognitive impairment.^36^ Still, the potential link between opioids and dementia is not completely understood, emphasizing the need for future studies to investigate opioids’ role in dementia development, particularly according to dementia etiology. This study highlights a potential long-acting influence of high opioid exposure unrelated to the strength of the opioid.

A population-based study found persistent pain to be associated with accelerated cognitive decline and dementia.^37^ However, the study did not account for use of analgesics; therefore, the authors hypothesized that the observed association could be mediated by opioid use.

We cannot rule out that our findings were influenced by confounding by indication. Still, the results among individuals with pain-intensive diagnoses indicate that the observed association between opioids and dementia is likely partly but not entirely explained by the hypothesized influence of chronic pain or the underlying conditions leading to pain.

Chronic noncancer pain presents a global public health challenge with both a high and increasing prevalence.^5,6^ Opioids are frequently used for such indications, and inappropriate prescribing has been reported in both Eastern and Western countries.^38^ Particularly, use of the weak opioid tramadol is widespread and increasing and may lead to long-term use of strong opioids.^28^ In our study, dementia rates remained elevated when restricting to weak opioids only. Therefore, we call for scientific attention and further research exploring the long-term brain safety of opioids and interventions aimed at reducing inappropriate opioid use.

Our study findings are generalizable to populations with comparable health care and demographics. The comprehensive Danish registers are a relevant data foundation for addressing the present research question, especially considering the relatively high and increasing use of prescription opioids in Denmark during the study period proximal to that of North America, as also seen in several other European countries such as Germany and Austria.^31,39^

Study strengths include a large nationwide sample with long follow-up, diminishing selection bias, and exposure assessment via redeemed prescriptions, hindering information bias. Highly valid dementia diagnoses were used and inclusion of several relevant confounding variables.^25^

### Limitations

This study had limitations. One limitation included unavailable information on opioid exposure before 1995, resulting in underestimating the amount of opioid use, thereby, potentially diluting the observed association. Considering reporting an overall positive association with dementia, we do not expect this limitation to have materially impacted the interpretation.

Another limitation was residual confounding, including potential confounding by indication cannot be ruled out in observational studies. We could not differentiate between opioid use and the underlying disorder leading to treatment. Nonetheless, tackling such potential confounding, associations remained stable for highest levels of opioid exposure among individuals with chronic noncancer pain and with exposure restricted to weak opioids. Severity and duration of chronic pain was unavailable, but sensitivity analyses suggested that pain was partially but not entirely responsible for the increased dementia risk. Potential residual confounding from unmeasured variables, including lifestyle-related factors such as alcohol use and/or alcohol use disorder or underregistered opioid addiction, cannot be ruled out because national Danish registers do not hold these data. Still, we reduced potential reverse causation bias with a 5-year lag-time window, and we were able to adjust for several known conditions associated with unhealthy lifestyle that may affect dementia risk. Additionally, influence from competing risk of death cannot be excluded (particularly in the oldest individuals aged above 90 years), although this was reduced by excluding individuals with cancer, opioid use disorder, and usage in terminal illness. Based on our findings of increased mortality rate among those with opioid use greater than 90 TSDs, we assume at worst an underestimation of the observed association with dementia. Thus, we do not expect this competing risk to have materially impacted the conclusions.

## Conclusions

This study found cumulative opioid exposure below 90 TSDs not to be uniformly associated with increased dementia risk, although some models found a statistically significant positive association with 31 to 90 TSDs. Exposure to more than 90 TSDs was associated with a slightly increased dementia risk. Chronic pain could partly explain associations but not entirely. For greater than 90 TSDs, associations overall persisted in individuals with chronic noncancer pain and individuals receiving weak opioids. While the clinical importance mainly relates to opioid use greater than 90 TSDs, the increased global opioid use over the past 2 decades and prevalence of chronic noncancer pain highlight the importance of exploring our findings further. Future studies are warranted to study opioid use and risk of dementia subtypes.

## Notes

Supplement 1. **eTable 1.** Diagnosis and Drug Codes **eTable 2.** Equianalgesic Ratio for Various Opioids and Formulations **eTable 3.** Opioid Formulations and Usage Among Opioid Users in the Matched Population **eFigure 1.** Adjusted Incidence Rate Ratios (IRR) and 95% Confidence Intervals (CI) of the Association Between User Status by Cumulative Use of Opioids and All-Cause Dementia According to Age at Index **eFigure 2.** Adjusted Incidence Rate Ratios (IRR) and 95% Confidence Intervals (CI) of the Association Between Cumulative Use of Opioid Antitussives and All-Cause Dementia According to Age at Index **eFigure 3.** Adjusted Incidence Rate Ratios (IRR) and 95% Confidence Intervals (CI) of the Association Between Cumulative Opioid Use and All-Cause Dementia According to Age at Index With One-Year Lag-Time **eFigure 4.** Adjusted Incidence Rate Ratios (IRR) and 95% Confidence Intervals (CI) of the Association Between Cumulative Opioid Use and All-Cause Dementia According to Age at Index With Covariates Defined at Baseline **eFigure 5.** Adjusted Mortality Rate Ratios (RR) and 95% Confidence Intervals (CI) of the Association Between Cumulative Opioid Use and Mortality According to Age at Index

Supplement 2.
Data Sharing Statement
