# Supplementary material for: Opioids and Dementia in the Danish Population
Source: JAMA Netw Open. 2024 Nov 19;7(11):e2445904. doi: 10.1001/jamanetworkopen.2024.45904 (PMC11577141; doi:10.1001/jamanetworkopen.2024.45904)
Supplement: Supplement 1. — eTable 1. Diagnosis and Drug Codes eTable 2. Equianalgesic Ratio for Various Opioids and Formulations eTable 3. Opioid Formulations and Usage Among Opioid Users in the Matched Population eFigure 1. Adjusted Incidence Rate Ratios (IRR) and 95% Confidence Intervals (CI) of the Association Between User Status by Cumulative Use of Opioids and All-Cause Dementia According to Age at Index eFigure 2. Adjusted Incidence Rate Ratios (IRR) and 95% Confidence Intervals (CI) of the Association Between Cumulative Use of Opioid Antitussives and All-Cause Dementia According to Age at Index eFigure 3. Adjusted Incidence Rate Ratios (IRR) and 95% Confidence Intervals (CI) of the Association Between Cumulative Opioid Use and All-Cause Dementia According to Age at Index With One-Year Lag-Time eFigure 4. Adjusted Incidence Rate Ratios (IRR) and 95% Confidence Intervals (CI) of the Association Between Cumulative Opioid Use and All-Cause Dementia According to Age at Index With Covariates Defined at Baseline eFigure 5. Adjusted Mortality Rate Ratios (RR) and 95% Confidence Intervals (CI) of the Association Between Cumulative Opioid Use and Mortality According to Age at Index [file jamanetwopen-e2445904-s001.pdf]

Supplemental Online Content

Pourhadi N, Janbek J, Gasse C, Laursen TM, Waldemar G, Jensen-Dahm C. Opioids and dementia in the Danish population. *JAMA Netw Open.* 2024;7(11):e2445904. doi:10.1001/jamanetworkopen.2024.45904

eTable 1. Diagnosis and Drug Codes

eTable 2. Equianalgesic Ratio for Various Opioids and Formulations

eTable 3. Opioid Formulations and Usage Among Opioid Users in the Matched Population

eFigure 1. Adjusted Incidence Rate Ratios (IRR) and 95% Confidence Intervals (CI) of the Association Between User Status by Cumulative Use of Opioids and All-Cause Dementia According to Age at Index

eFigure 2. Adjusted Incidence Rate Ratios (IRR) and 95% Confidence Intervals (CI) of the Association Between Cumulative Use of Opioid Antitussives and All-Cause Dementia According to Age at Index

eFigure 3. Adjusted Incidence Rate Ratios (IRR) and 95% Confidence Intervals (CI) of the Association Between Cumulative Opioid Use and All-Cause Dementia According to Age at Index With One-Year Lag-Time

eFigure 4. Adjusted Incidence Rate Ratios (IRR) and 95% Confidence Intervals (CI) of the Association Between Cumulative Opioid Use and All-Cause Dementia According to Age at Index With Covariates Defined at Baseline

eFigure 5. Adjusted Mortality Rate Ratios (RR) and 95% Confidence Intervals (CI) of the Association Between Cumulative Opioid Use and Mortality According to Age at Index

This supplemental material has been provided by the authors to give readers additional information about their work.

© 2024 Pourhadi N et al. *JAMA Network Open.*

eTable 1. Diagnosis and drug codes

<table>
<tr><th colspan="2" rowspan="2">Variable</th><th rowspan="2">Data source</th><th rowspan="2">Period of data availability</th><th rowspan="2">Classification system</th><th colspan="2">Definition</th></tr>
<tr><th>ICD 8 & 10</th><th>ATC code</th></tr>
<tr><td colspan="2">Dementia</td><td>The National Patient Register, The Danish Psychiatric Central Research Register & The National Prescription Register</td><td>1977-2020 & 1995-2020</td><td>ICD-8, ICD-10, & ATC</td><td>290,
F00, F01, F02, F03, G30, G31.8-9</td><td>N06D</td></tr>
<tr><td colspan="2">Opioid use</td><td>The National Prescription Register</td><td>1995-2020</td><td>ATC</td><td>-</td><td>N02A, R05DA, R05FA02</td></tr>
<tr><td colspan="2">Opioid addiction or opioid use in terminal illness</td><td>The National Patient Register, The Danish Psychiatric Central Research Register & The National Prescription Register</td><td>1977-2020 & 1995-2020</td><td>ICD-8, ICD-10, & ATC</td><td>30409, 30419, F112-114</td><td>N02A+injection administration, N02AE01+ sublingual administration, N07BC</td></tr>
<tr><td colspan="2">Cancer (except non-melanoma skin cancer)</td><td>The National Patient Register</td><td>1977-2020</td><td>ICD-8 & ICD-10</td><td>14-20 (except 173),
C (except C44)</td><td>-</td></tr>
<tr><td rowspan="3">Cardio-vascular disease</td><td>Stroke</td><td>The National Patient Register</td><td>1977-2020</td><td>ICD-8 & ICD-10</td><td>430-434, 436, I60-I64, I69</td><td>-</td></tr>
<tr><td>Ischemic heart disease & acute myocardial infarction</td><td>The National Patient Register & The National Prescription Register</td><td>1977-2020 & 1995-2020</td><td>ICD-8, ICD-10, & ATC</td><td>410.99,
I20-I25</td><td>C01DA</td></tr>
<tr><td>Oral antithrombotic medication & anticoagulants</td><td>The National Prescription Register</td><td>1995-2020</td><td>ATC</td><td>-</td><td>B01A</td></tr>
<tr><td colspan="2">Diabetes Mellitus</td><td>The National Patient Register & The National Prescription Register</td><td>1977-2020 & 1995-2020</td><td>ICD-8, ICD-10, & ATC</td><td>249-250,
E10-E14</td><td>A10</td></tr>
<tr><td colspan="2">Hypertension</td><td>The National Patient Register & The National Prescription Register</td><td>1977-2020 & 1995-2020</td><td>ICD-8, ICD-10, & ATC</td><td>400-404, 410.09, 411.09, 412.09, 413.09, 414.09, 435.09, 437.00, 437.01, 437.08, 437.09, 438.09, I10-I13, I15</td><td>C02-C04, C07-C09</td></tr>
<tr><td colspan="2">Dyslipidemia</td><td>The National Patient Register & The National Prescription Register</td><td>1977-2020 & 1995-2020</td><td>ICD-8, ICD-10, & ATC</td><td>279.00,
E78.0</td><td>C10</td></tr>
<tr><td colspan="2">Education</td><td></td><td>1980-2020</td><td></td><th colspan="2">Definition</th></tr>
</table>

	Danish Education Registers		Highest obtained education - International Standard Classification of Education	Elementary/secondary school; Vocational education; University education	
**Variable**	**Data source**	**Period of data availability**	**Classification system**	**Definition**	
				**ICD 8 & 10**	**ATC code**
**Charlson Comorbidity Index (modified)**					
Congestive heart failure	The National Patient Register	1977-2020	ICD-8 & ICD-10	42709, 42710, 42711, 42719, 42899, 78249, I50, I110, I130, I132	
Peripheral vascular disease				440, 441, 442, 443, 444, 445, I70, I71, I72, I73, I74, I77	
Chronic pulmonary disease				490-493, 515-518, J40-J47, J60-J67, J684, J701, J703, J841, J920, J961, J982, J983	
Connective tissue disease				712, 716, 734, 446, 13599, M05, M06, M08, M09, M30, M31, M32, M33, M34, M35, M36, D86	
Ulcer disease				53091, 53098, 531-534, K221, K25-K28	
Mild liver disease				571, 57301, 57304, B18, K700-K703, K709, K71, K73, K74, K760	
Hemiplegia				344, G81, G82	
Moderate to severe renal disease				403, 404, 580-585, 59009, 59319, 75310-75319, 792, I12, I13, N00-N05, N07, N11, N14, N17-N19, Q61	
Moderate to severe liver disease				07000, 07002, 07004, 07006, 07008, 57300, 45600-45609, B150, B160, B162, B190, K704, K72, K766, I85	
AIDS				07983, B21-B24	
**Chronic non-cancer pain**					
Pain-intensive back diagnoses/intervertebral disc pain	The National Patient Register	1977-2020	ICD-8 & ICD-10	M43, M45, M46, M48-51, M81, M82, 713, 72309, 725, 839	
Arthritic pain				M05-08, M10-19, M23-25, M36, M77, R26, 27400, 27401, 7120-7123, 714, 715	
Posttraumatic fracture pain				S12, S22, S32, S42, S43, S53, T02, T08, T91, 805-807, 811, 812, 831, 832, 8490, 8491	
Neuropathic pain				M792, M890, G50, G52-64, G82, G97, R29, 24903, 25003, 30391, 34302, 34303, 35401, 35408, 35409, 35100, 35101, 35109	

© 2024 Pourhadi N et al. *JAMA Network Open.*

eTable 2. Equianalgesic ratio for various opioids and formulations

Drug	ATC-codes	Administration	Equianalgesic ratio*
**Analgesic strong opioids**			
**Morphine**	N02AA01, N02AA04, N02AG01	Oral/rectal	1
**Morphine**	N02AA01, N02AA04	Parenteral	3
**Oxycodone**	N02AA05, N02AA55	Oral	1.5
**Buprenorphine**	N02AE01	Sublingual	50
**Buprenorphine**	N02AE01	Transdermal	110
**Fentanyl**	N02AB03	Sublingual	50
**Fentanyl**	N02AB03	Transdermal/nasal/oral	100
**Hydromorphone**	N02AA03	Oral	6
**Ketobemidone**	N02AB01, N02AG02	Oral/rectal	1
**Ketobemidone**	N02AG02	Parenteral	3
**Dextromoramide**	N02AC01	Oral	3
**Analgesic weak opioids**			
**Pethidine**	N02AB02	Oral/rectal	0.1
**Codeine**	N02AJ06, N02AJ07	Oral	0.1
**Tramadol**	N02AX02	Oral/rectal	0.2
**Tapantadol**	N02AX06	Oral	0.4
**Dextropropoxyphene**	N02AC04	Oral	0.15
**Pentazocine**	N02AD01	Oral/rectal	0.5
**Antitussives**			
**Codeine**	R05DA04, R05FA02	Oral	0.1
**Opium**	R05FA02	Oral	1
**Dextromethorphan**	R05DA09	Oral	1
**Ethylmorphine**	R05DA01	Oral	0.1
**Noscapine**	R05DA07	Oral	0.1

* Svendsen K, Borchgrevink P, Fredheim O, Hamunen K, Mellbye A, Dale O. Choosing the unit of measurement counts: The use of oral morphine equivalents in studies of opioid consumption is a useful addition to defined daily doses. *Palliat Med* 2011; 25: 725–32.

Calculation of Total Standardized Dose (TSD)

The oral morphine equivalent dose (OMEQ) is a measurement unit that considers the analgesic potency of each opioid according to its type and mode of administration by using the equianalgesic ratio (EAR). The specific OMEQ is calculated by multiplying the opioid dose with its respective EAR:

$$OMEQ = [Opioid\ dose] * EAR$$

One TSD is defined as 30 mg oral morphine per day.

TSD is calculated by dividing the total amount of OMEQ (in milligrams) by 30 mg:

$$TSD = \frac{OMEQ(mg)}{30\ mg}$$

© 2024 Pourhadi N et al. *JAMA Network Open.*

eTable 3. Opioid formulations and usage among opioid users in the matched population

Drug	ATC-codes	Mode of administration	Most frequently used dosage per unit	EAR[a]	Users, No. (%)[b]	Used TSDs, No. (%)[c]
**Analgesic strong opioids**						
**Morphine**	N02AA01 N02AA04 N02AG01	Oral	10 mg	1	20,093 (6.7)	10,419,479 (12.6)
		Rectal	10 mg	1	677 (0.2)	28,661 (<0.1)
**Oxycodone**	N02AA05 N02AA55	Oral	5 mg	1.5	22,395 (7.5)	6,424,820 (7.8)
**Ketobemidone**	N02AB01 N02AG02	Oral	5 mg	1	24,129 (8.0)	3,286,200 (4.0)
		Rectal	10 mg	1	3944 (1.3)	223,030 (0.3)
**Fentanyl**	N02AB03	Oral	0.6 mg	100	<20 (<0.1)	<100 (<0.1)
		Transdermal	25 µg/hour	100	2474 (0.8)	3,497,676 (4.2)
**Buprenorphine**	N02AE01	Transdermal	5 µg/hour	110	4644 (1.5)	1,488,005 (1.8)
**Hydromorphone**	N02AA03	Oral	8 mg	6	65 (<0.1)	48,400 (0.1)
**Dextromoramide**	N02AC01	Oral	5 mg	3	45 (<0.1)	3,500 (<0.1)
**Analgesic weak opioids**						
**Tramadol**	N02AX02	Oral	50 mg	0.2	147,611 (49.2)	38,382,494 (46.4)
		Rectal	100 mg	0.2	1061 (0.4)	65,413 (0.1)
**Codeine**	N02AJ06 N02AJ07	Oral	30.6 mg	0.1	57,584 (19.2)	2,000,137 (2.4)
**Dextropropoxyphene**	N02AC04	Oral	65 mg	0.15	7863 (2.6)	4,810,093 (5.8)
**Pethidine**	N02AB02	Oral	25 mg	0.1	1959 (0.7)	110,412 (0.1)
		Rectal	100 mg	0.1	2361 (0.8)	424,508 (0.5)
**Tapantadol**	N02AX06	Oral	50 mg	0.4	138 (<0.1)	45,600 (0.1)
**Pentazocine**	N02AD01	Oral	50 mg	0.5	417 (0.1)	302,533 (0.4)
		Rectal	50 mg	0.5	28 (<0.1)	17,508 (<0.1)
**Antitussives**						
**Opium**	R05FA02	Oral	1.7 mg	1	146,843 (48.9)	7,012,634 (8.5)
**Codeine**	R05DA04 R05FA02	Oral	25 mg	0.1	99,626 (33.2)	3,908,259 (4.7)
**Dextromethorphan**	R05DA09	Oral	3 mg	1	8335 (2.8)	239,530 (0.3)
**Ethylmorphine**	R05DA01	Oral	1.7 mg	0.1	4317 (1.4)	7938 (<0.1)
**Noscapine**	R05DA07	Oral	25 mg	0.1	2244 (0.7)	6735 (<0.1)

[a] Equianalgesic ratio
[b] The total number of unique opioid users was n=300,124
[c] The total number of used TSDs was n=82,753,573

eFigure 1. Adjusted incidence rate ratios (IRR) and 95% confidence intervals (CI) of the association between user status by cumulative use of opioids and all-cause dementia according to age at index.

User status, cumulative opioid use (TSDs)	Cases (n, %)	Controls (n, %)	IRR(CI_{95})
60–69 years at index			
No use	5,352 (39.0)	31,118 (45.3)	Reference
Last treatment day within 5 yr of index, <90 TSDs	3,110 (22.6)	15,782 (23.0)	0.97 (0.92–1.02)
Last treatment day within 5 yr of index, 90+ TSDs	2,617 (19.0)	8,013 (11.7)	1.29 (1.22–1.37)
Last treatment day 5+ yr before index, <90 TSDs	2,380 (17.3)	12,864 (18.7)	1.00 (0.95–1.06)
Last treatment day 5+ yr before index, 90+ TSDs	279 (2.0)	913 (1.3)	1.45 (1.26–1.67)
70–79 years at index			
No use	14,549 (34.0)	81,468 (38.1)	Reference
Last treatment day within 5 yr of index, <90 TSDs	9,673 (22.6)	51,728 (24.2)	0.94 (0.91–0.97)
Last treatment day within 5 yr of index, 90+ TSDs	9,794 (22.9)	36,304 (17.0)	1.23 (1.19–1.27)
Last treatment day 5+ yr before index, <90 TSDs	7,711 (18.0)	40,287 (18.8)	1.02 (0.99–1.06)
Last treatment day 5+ yr before index, 90+ TSDs	1,030 (2.4)	3,998 (1.9)	1.28 (1.19–1.37)
80–89 years at index			
No use	8,741 (25.0)	48,179 (27.6)	Reference
Last treatment day within 5 yr of index, <90 TSDs	7,649 (21.9)	40,411 (23.1)	0.98 (0.95–1.01)
Last treatment day within 5 yr of index, 90+ TSDs	10,181 (29.1)	44,568 (25.5)	1.13 (1.10–1.17)
Last treatment day 5+ yr before index, <90 TSDs	7,158 (20.5)	36,254 (20.8)	1.05 (1.02–1.09)
Last treatment day 5+ yr before index, 90+ TSDs	1,200 (3.4)	5,233 (3.0)	1.19 (1.11–1.27)
90+ years at index			
No use	407 (18.4)	2,181 (19.7)	Reference
Last treatment day within 5 yr of index, <90 TSDs	450 (20.3)	2,205 (19.9)	1.06 (0.91–1.23)
Last treatment day within 5 yr of index, 90+ TSDs	753 (34.0)	3,554 (32.1)	1.08 (0.94–1.24)
Last treatment day 5+ yr before index, <90 TSDs	494 (22.3)	2,547 (23.0)	1.02 (0.89–1.18)
Last treatment day 5+ yr before index, 90+ TSDs	110 (5.0)	583 (5.3)	0.99 (0.78–1.25)

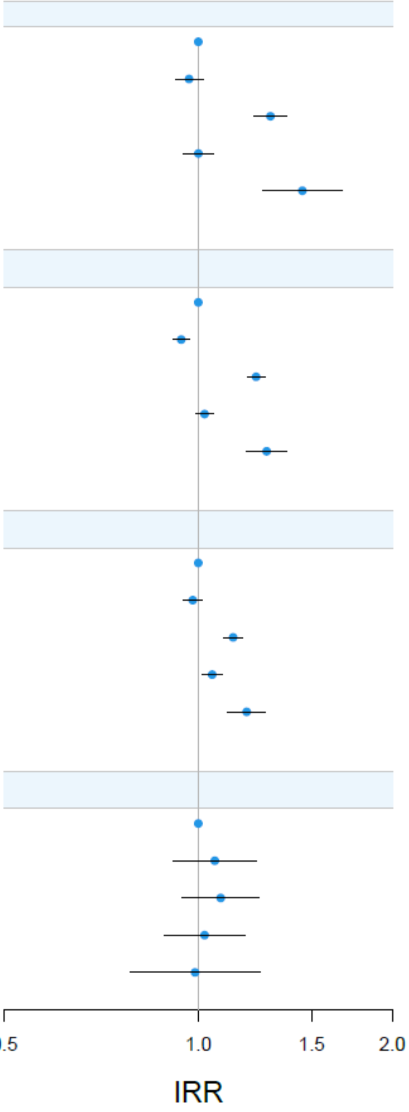

Footnote: Adjusted for educational level, cardiovascular disease, diabetes, hypertension, dyslipidemia, and CCI score. No lag-time window applied.

TSD = Total standardized daily dose.

© 2024 Pourhadi N et al. *JAMA Network Open.*

eFigure 2. Adjusted incidence rate ratios (IRR) and 95% confidence intervals (CI) of the association between cumulative use of opioid antitussives and all-cause dementia according to age at index.

Cumulative opioid use (TSDs)	Cases (n, %)	Controls (n, %)	IRR(CI_{95})
60–69 years at index			
No use	5,887 (77.9)	29,497 (78.1)	Reference
Ever use	1,667 (22.1)	8,273 (21.9)	0.97 (0.92–1.04)
0–30	1,226 (16.2)	6,222 (16.5)	0.96 (0.89–1.03)
31–90	336 (4.4)	1,652 (4.4)	0.99 (0.87–1.11)
91–200	67 (0.9)	308 (0.8)	0.99 (0.76–1.30)
>200	38 (0.5)	91 (0.2)	1.91 (1.30–2.81)
70–79 years at index			
No use	16,055 (74.3)	81,005 (75.0)	Reference
Ever use	5,553 (25.7)	27,035 (25.0)	1.00 (0.97–1.04)
0–30	3,849 (17.8)	19,249 (17.8)	0.98 (0.95–1.02)
31–90	1,268 (5.9)	5,958 (5.5)	1.03 (0.97–1.10)
91–200	300 (1.4)	1,356 (1.3)	1.06 (0.94–1.21)
>200	136 (0.6)	472 (0.4)	1.36 (1.12–1.65)
80–89 years at index			
No use	9,470 (68.9)	47,522 (69.2)	Reference
Ever use	4,271 (31.1)	21,183 (30.8)	0.99 (0.96–1.04)
0–30	2,901 (21.1)	14,399 (21.0)	1.00 (0.95–1.04)
31–90	984 (7.2)	4,980 (7.2)	0.97 (0.90–1.04)
91–200	276 (2.0)	1,273 (1.9)	1.06 (0.93–1.21)
>200	110 (0.8)	531 (0.8)	1.02 (0.83–1.25)
90+ years at index			
No use	448 (62.1)	2,330 (64.6)	Reference
Ever use	273 (37.9)	1,275 (35.4)	1.10 (0.93–1.30)
0–30	168 (23.3)	852 (23.6)	1.01 (0.83–1.23)
31–90	79 (11.0)	297 (8.2)	1.37 (1.05–1.80)
91–200	19 (2.6)	90 (2.5)	1.08 (0.65–1.80)
>200	7 (1.0)	36 (1.0)	0.98 (0.44–2.21)

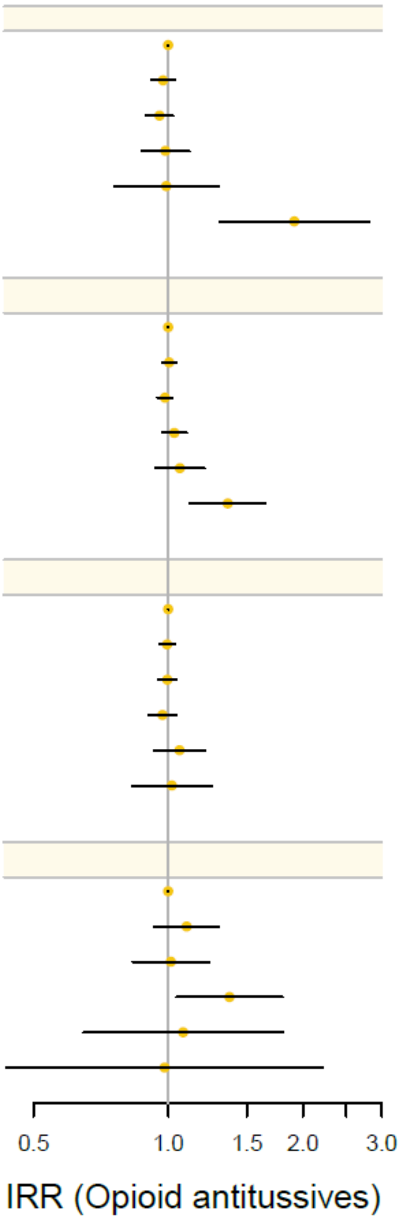

Footnote: Adjusted for educational level, cardiovascular disease, diabetes, hypertension, dyslipidemia, and CCI score. Five-year lag-time window applied.

TSD = Total standardized daily dose. Further stratification to >500 TSDs was not possible due to low number of cases.

© 2024 Pourhadi N et al. *JAMA Network Open.*

eFigure 3. Adjusted incidence rate ratios (IRR) and 95% confidence intervals (CI) of the association between cumulative opioid use and all-cause dementia according to age at index with one-year lag-time.

Cumulative opioid use (TSDs)	Cases (n, %)	Controls (n, %)	IRR(CI_{95})
60–69 years at index			
No use	5,765 (42.0)	32,603 (47.5)	Reference
Ever use	7,973 (58.0)	36,087 (52.5)	1.08 (1.04–1.12)
0–30	3,522 (25.6)	19,128 (27.8)	0.97 (0.93–1.02)
31–90	1,901 (13.8)	8,906 (13.0)	1.06 (1.00–1.13)
91–200	854 (6.2)	3,410 (5.0)	1.17 (1.07–1.27)
201–500	647 (4.7)	1,970 (2.9)	1.42 (1.29–1.57)
>500	1,049 (7.6)	2,673 (3.9)	1.60 (1.47–1.74)
70–79 years at index			
No use	15,853 (37.1)	86,544 (40.5)	Reference
Ever use	26,904 (62.9)	127,241 (59.5)	1.07 (1.04–1.09)
0–30	10,807 (25.3)	58,019 (27.1)	0.98 (0.96–1.01)
31–90	6,561 (15.3)	32,900 (15.4)	1.02 (0.99–1.06)
91–200	3,361 (7.9)	14,874 (7.0)	1.13 (1.08–1.17)
201–500	2,397 (5.6)	9,480 (4.4)	1.23 (1.17–1.29)
>500	3,778 (8.8)	11,968 (5.6)	1.50 (1.44–1.56)
80–89 years at index			
No use	9,847 (28.2)	51,634 (29.6)	Reference
Ever use	25,082 (71.8)	123,011 (70.4)	1.04 (1.01–1.06)
0–30	8,817 (25.2)	45,784 (26.2)	1.00 (0.96–1.03)
31–90	6,161 (17.6)	31,416 (18.0)	1.00 (0.97–1.04)
91–200	3,403 (9.7)	16,999 (9.7)	1.02 (0.97–1.06)
201–500	2,712 (7.8)	12,077 (6.9)	1.14 (1.08–1.19)
>500	3,989 (11.4)	16,735 (9.6)	1.20 (1.15–1.26)
90+ years at index			
No use	457 (20.6)	2,345 (21.2)	Reference
Ever use	1,757 (79.4)	8,725 (78.8)	1.04 (0.93–1.17)
0–30	531 (24.0)	2,752 (24.9)	0.99 (0.86–1.14)
31–90	453 (20.5)	2,097 (18.9)	1.11 (0.96–1.29)
91–200	256 (11.6)	1,336 (12.1)	0.99 (0.84–1.18)
201–500	196 (8.9)	1,017 (9.2)	1.00 (0.83–1.21)
>500	321 (14.5)	1,523 (13.8)	1.10 (0.94–1.30)

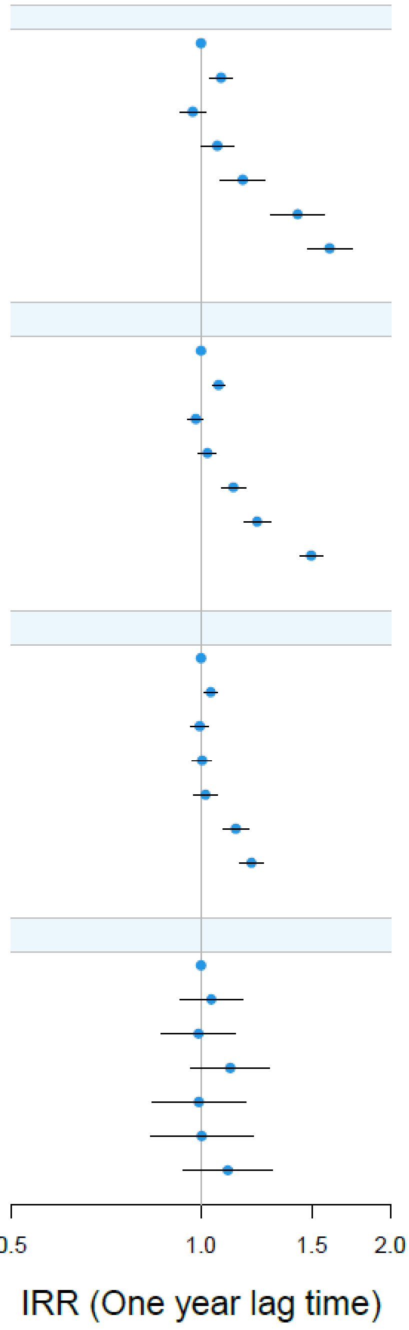

Footnote: Adjusted for educational level, cardiovascular disease, diabetes, hypertension, dyslipidemia, and CCI score. One-year lag-time window applied.

TSD = Total standardized daily dose

© 2024 Pourhadi N et al. *JAMA Network Open.*

eFigure 4. Adjusted incidence rate ratios (IRR) and 95% confidence intervals (CI) of the association between cumulative opioid use and all-cause dementia according to age at index with covariates defined at baseline.

Cumulative opioid use (TSDs)	Cases (n, %)	Controls (n, %)	IRR(CI_{95})
60–69 years at index			
No use	7,343 (53.5)	39,974 (58.2)	Reference
Ever use	6,395 (46.5)	28,716 (41.8)	1.09 (1.05–1.13)
0–30	3,312 (24.1)	16,907 (24.6)	1.01 (0.97–1.06)
31–90	1,413 (10.3)	6,592 (9.6)	1.05 (0.99–1.13)
91–200	611 (4.4)	2,233 (3.3)	1.27 (1.16–1.40)
201–500	428 (3.1)	1,306 (1.9)	1.43 (1.27–1.60)
>500	631 (4.6)	1,678 (2.4)	1.55 (1.41–1.72)
70–79 years at index			
No use	20,597 (48.2)	109,909 (51.4)	Reference
Ever use	22,160 (51.8)	103,876 (48.6)	1.09 (1.07–1.12)
0–30	10,529 (24.6)	54,327 (25.4)	1.01 (0.99–1.04)
31–90	5,396 (12.6)	26,004 (12.2)	1.07 (1.04–1.11)
91–200	2,404 (5.6)	10,294 (4.8)	1.19 (1.13–1.24)
201–500	1,603 (3.7)	6,197 (2.9)	1.29 (1.21–1.36)
>500	2,228 (5.2)	7,054 (3.3)	1.53 (1.45–1.61)
80–89 years at index			
No use	12,866 (36.8)	67,292 (38.5)	Reference
Ever use	22,063 (63.2)	107,353 (61.5)	1.06 (1.03–1.08)
0–30	9,392 (26.9)	48,148 (27.6)	1.01 (0.98–1.04)
31–90	5,580 (16.0)	27,628 (15.8)	1.04 (1.01–1.08)
91–200	2,748 (7.9)	12,883 (7.4)	1.10 (1.05–1.15)
201–500	1,890 (5.4)	8,486 (4.9)	1.14 (1.08–1.21)
>500	2,453 (7.0)	10,208 (5.8)	1.22 (1.17–1.29)
90+ years at index			
No use	600 (27.1)	3,123 (28.2)	Reference
Ever use	1,614 (72.9)	7,947 (71.8)	1.05 (0.95–1.17)
0–30	585 (26.4)	3,017 (27.3)	1.00 (0.89–1.14)
31–90	440 (19.9)	1,997 (18.0)	1.14 (0.99–1.31)
91–200	216 (9.8)	1,129 (10.2)	0.99 (0.83–1.18)
201–500	170 (7.7)	832 (7.5)	1.05 (0.87–1.27)
>500	203 (9.2)	972 (8.8)	1.09 (0.91–1.30)

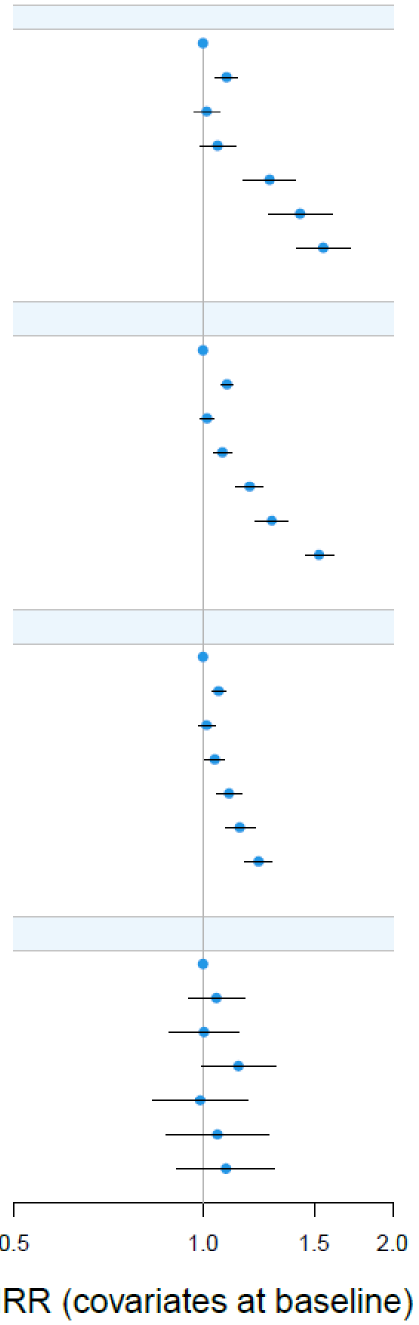

Footnote: Adjusted for educational level, cardiovascular disease, diabetes, hypertension, dyslipidemia, and CCI score at baseline. Five-year lag-time window applied.

TSD = Total standardized daily dose

© 2024 Pourhadi N et al. *JAMA Network Open.*

eFigure 5. Adjusted mortality rate ratios (RR) and 95% confidence intervals (CI) of the association between cumulative opioid use and mortality according to age at index.

Cumulative opioid use (TSDs)	Died (n, %)	Controls (n, %)	Mortality RR(CI_{95})
60−69 years at index			
No use	37,360 (54.4)	211,851 (61.6)	Reference
Ever use	31,372 (45.6)	131,809 (38.4)	1.04 (1.02−1.06)
0−30	14,136 (20.6)	79,386 (23.1)	0.89 (0.87−0.91)
31−90	6,729 (9.8)	29,672 (8.6)	0.99 (0.96−1.03)
91−200	3,297 (4.8)	9,999 (2.9)	1.26 (1.20−1.32)
201−500	2,529 (3.7)	5,763 (1.7)	1.51 (1.43−1.59)
>500	4,681 (6.8)	6,989 (2.0)	2.10 (2.01−2.20)
70−79 years at index			
No use	50,112 (48.6)	285,736 (55.4)	Reference
Ever use	52,973 (51.4)	229,689 (44.6)	1.04 (1.02−1.06)
0−30	22,077 (21.4)	124,593 (24.2)	0.90 (0.88−0.92)
31−90	11,824 (11.5)	55,738 (10.8)	0.98 (0.96−1.00)
91−200	6,114 (5.9)	21,804 (4.2)	1.17 (1.13−1.21)
201−500	4,868 (4.7)	12,910 (2.5)	1.42 (1.37−1.47)
>500	8,090 (7.8)	14,644 (2.8)	1.95 (1.89−2.01)
80−89 years at index			
No use	23,482 (34.2)	135,396 (39.5)	Reference
Ever use	45,117 (65.8)	207,599 (60.5)	1.04 (1.02−1.06)
0−30	16,701 (24.3)	94,331 (27.5)	0.92 (0.90−0.94)
31−90	10,581 (15.4)	53,815 (15.7)	0.97 (0.94−0.99)
91−200	5,807 (8.5)	24,934 (7.3)	1.08 (1.04−1.11)
201−500	4,726 (6.9)	15,896 (4.6)	1.29 (1.24−1.34)
>500	7,302 (10.6)	18,623 (5.4)	1.65 (1.60−1.70)
90+ years at index			
No use	2,473 (26.2)	13,617 (28.9)	Reference
Ever use	6,965 (73.8)	33,573 (71.1)	1.03 (0.98−1.09)
0−30	2,335 (24.7)	12,789 (27.1)	0.96 (0.90−1.02)
31−90	1,746 (18.5)	8,985 (19.0)	0.98 (0.92−1.05)
91−200	1,008 (10.7)	4,751 (10.1)	1.04 (0.95−1.13)
201−500	728 (7.7)	3,092 (6.6)	1.13 (1.03−1.24)
>500	1,148 (12.2)	3,956 (8.4)	1.38 (1.27−1.49)

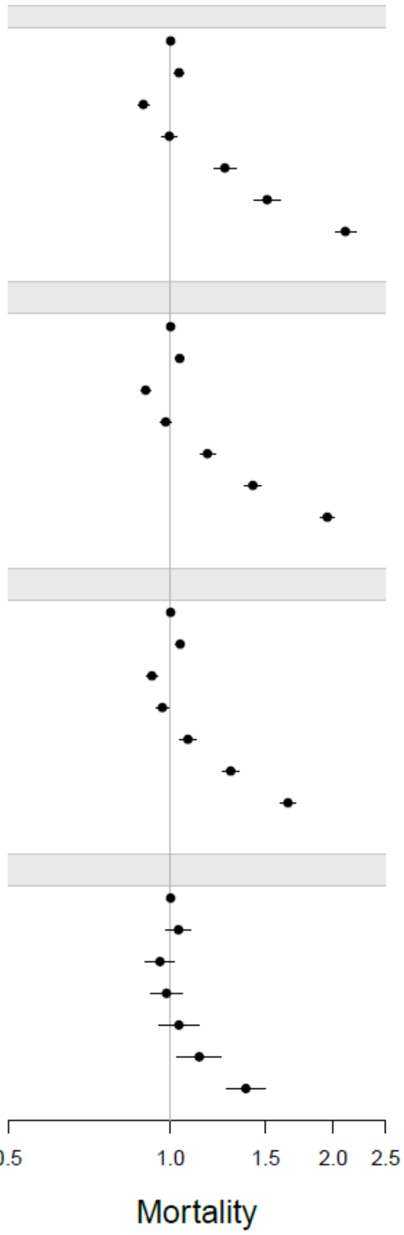

Footnote: Adjusted for educational level, cardiovascular disease, diabetes, hypertension, dyslipidemia, and CCI score. Five-year lag-time window applied.

TSD = Total standardized daily dose

© 2024 Pourhadi N et al. *JAMA Network Open.*
