# Supplementary material for: Opioids and Dementia in the Danish Population
Source: JAMA Netw Open. 2024 Nov 19;7(11):e2445904. doi: 10.1001/jamanetworkopen.2024.45904 (PMC11577141; doi:10.1001/jamanetworkopen.2024.45904)
Supplement: Supplement 2. — Data Sharing Statement [file jamanetwopen-e2445904-s002.pdf]

Data Sharing Statement

Pourhadi. Opioids and Dementia in the Danish Population. *JAMA Netw Open*. Published November 19, 2024. doi:10.1001/jamanetworkopen.2024.45904

Data
Data available: No

Additional Information
Explanation for why data not available: This study was based on raw data derived from the national Danish registers only available with approval from the Danish Health Data Board and the Danish Data Protection Agency. Since the data was accessible on individual level, data sharing is restricted by the General Data Protection Regulation (GDPR) of European Union (EU) law.
